# Multiple expressed MHC class II loci in salmonids; details of one non-classical region in Atlantic salmon (*Salmo salar*)

**DOI:** 10.1186/1471-2164-9-193

**Published:** 2008-04-28

**Authors:** Håvard Harstad, Morten F Lukacs, Hege G Bakke, Unni Grimholt

**Affiliations:** 1 Department of Basic Science and Aquatic Medicine, Norwegian School of Veterinary Science, Oslo, Norway

## Abstract

**Background:**

In teleosts, the Major Histocompatibility Complex (MHC) class I and class II molecules reside on different linkage groups as opposed to tetrapods and shark, where the class I and class II genes reside in one genomic region. Several teleost MHC class I regions have been sequenced and show varying number of class I genes. Salmonids have one major expressed MHC class I locus (UBA) in addition to varying numbers of non-classical genes. Two other more distant lineages are also identifyed denoted L and ZE. For class II, only one major expressed class II alpha (DAA) and beta (DAB) gene has been identified in salmonids so far.

**Results:**

We sequenced a genomic region of 211 kb encompassing divergent MHC class II alpha (*Sasa-DBA*) and beta (*Sasa-DBB*) genes in addition to NRGN, TIPRL, TBCEL and TECTA. The region was not linked to the classical class II genes and had some synteny to genomic regions from other teleosts. Two additional divergent and expressed class II sequences denoted DCA and DDA were also identified in both salmon and trout. Expression patterns and lack of polymorphism make these genes non-classical class II analogues. *Sasa-DBB*, *Sasa-DCA *and *Sasa-DDA *had highest expression levels in liver, hindgut and spleen respectively, suggestive of distinctive functions in these tissues. Phylogenetic studies revealed more yet undescribed divergent expressed MHC class II molecules also in other teleosts.

**Conclusion:**

We have characterised one genomic region containing expressed non-classical MHC class II genes in addition to four other genes not involved in immune function. Salmonids contain at least two expressed MHC class II beta genes and four expressed MHC class II alpha genes with properties suggestive of new functions for MHC class II in vertebrates. Collectively, our data suggest that the class II is worthy of more elaborate studies also in other teleost species.

## Background

Teleost fish are the largest group of vertebrates comprising almost half of the total living vertebrates. In tetrapods and sharks the major histocompatibility genes are linked in a complex on a single chromosome [1]. In all teleosts studied so far, including salmonids, the MHC class I and class II regions reside on different linkage groups [2-4]. Extensive studies of the genomic class I region has been conducted in several fish species including rainbow trout and Atlantic salmon [5-9]. The Atlantic salmon and rainbow trout genomes encode one major MHC class I locus designated *UBA *with additional non-classical MHC class I genes in two duplicated MHC class I regions [7,9]. Both regions also harbour genes involved in the antigen presentation pathway, including proteosome subunits and the transporter for antigen processing. These genes all reside in the class II region in mammals [10].

Genomic class II regions are described in detail in zebrafish [11] and stickleback [12]. In zebrafish, one class II alpha locus and a number of class II beta loci residing on two different linkage groups have previously been identified, where only the *DAA *and *DAB *loci are known to be expressed [13]. Analysis of the functional class II region in zebrafish showed close linkage of the *DAA *and *DAB *loci on chromosome 8, but lack of other genes residing in the human MHC region [14]. In stickleback, a 99.5 kb genomic segment contained a tandem duplicate of expressed MHC class II alpha and beta genes, designated *Gaac-DAA/DAB *and *Gaac-DBA/DBB*. In Atlantic salmon, the major MHC class II alpha and beta genes are designated *DAA *and *DAB *respectively. They are closely linked and cosegregate as functional haplotypes [3,15-18]. Both the class II alpha as well as the class II beta chains have polymorphic alpha 1 and beta 1 domains [15,19], although much less polymorphic than the class I alpha 1 domain [3].

In humans, there are three classical expressed class II loci denoted HLA-DP, DQ and DR. Two additional nonclassical expressed MHC class II molecules, HLA-DM and HLA-DO, are found to control the composition of the peptide repertoire displayed by MHC class II molecules on the cell surface of antigen presenting cells [20]. The nonclassical class II molecule HLA-DM regulates unloading of CLIP and loading of peptide onto classical MHC class II molecules [21]. CLIP is derived from the invariant chain (Ii) and functions as a chaperone for class II molecules as it mediates and maintains correct assembly of alpha beta dimers. HLA-DM also serves as a peptide editor in early endocytic compartments [22]. HLA-DO preferentially promotes peptide loading of MHC class II molecules that are dependent on the chaperone activity of DM, and influences editing in a positive way for some peptides and negatively for others [23]. In terms of polymorphism, HLA-DO and HLA-DM are generally unpolymorphic in contrast to the three classical expressed loci HLA-DP, DQ and DR [24,25].

Reports on non-classical MHC class II molecules in teleosts have so far been scarce, although sequences with low identity to their classical DAB counterparts have been described in *Xiphophorus *fishes and in the guppy *Poecilia reticulata *[26]. The aim of this study was to analyse the MHC class II situation in Atlantic salmon through low stringency screening of available BAC libraries using classical *DAA *and *DAB *probes. Here we describe the genomic organization and expression patterns of two Atlantic salmon nonclassical MHC class II genes. Through phylogenetic and comparative analyses we also uncover additional nonclassical MHC class II molecules.

## Results and discussion

An Atlantic salmon BAC library [27] screened at low stringency with radioactive labeled probes for MHC class IIα (*DAA*) and class IIβ (*DAB*) hybridized to 20 BAC clones (Table 1). The clones were ordered into one DAA and one DAB contig by restriction fragment analysis and southern hybridization. Some DAB positive BACs contained unstable inserts, displaying several deletions during restriction mapping. GRASP *Hin*dIII fingerprint information [28] confirmed our DAA contig, while DAB clones were placed in different contigs or identified as singletons potentially due to their unstable nature.

**Table 1:** Atlantic salmon BAC library screen.

**Probe(s)**	**No. of positive clones**	**BAC clone identities**
DAA	8	139H17, 311J12, 327D01, 430J21, 485N02, 579A17, 630N19, 650J16
DAB	12	113K11, 121O12, 332F21, 416L17, 417A07, 429J19, 525D04, 537K01, 612D01, 619A16, 642F05, 667J06

Positive Atlantic salmon BAC clones from initial library screen using probes for *Sasa-DAA *and *Sasa-DAB*.

To decipher between classical DAA/DAB-positive BACs and other potential class II BACs we tested both genomic DNA from the library fish as well as MHC class II positive BACs for presence of a class II alpha minisatellite marker residing in the 3'UTR of *Sasa-DAA*. Classical MHC class II alpha and beta alleles cosegregate as haplotypes in Atlantic salmon [15] and most haplotypes show a stable linkage between haplotype and class II alpha 3'UTR marker. The BAC library fish was homozygous for a marker previously shown to segregate with the DAA*0101-DAB*0801 haplotype [15]. None of the DAA or DAB positive BACs were positive for the marker, suggesting they potentially represented new MHC class II loci while the classical MHC class II region was not present in the BAC library.

Due to the confusing fingerprints analysis and deletions in the DAB positive BACs, they were not considered for full-sequencing. Among the DAA positive BACs, clone 630N19 had a centered DAA-positive fragment (data not shown), and was thus subjected to shotgun sequencing. Readings were assembled with a redundancy greater than 10 over 211,190 bp [GenBank: EU008541]. One gap in the BAC clone 630N19 sequence still remains due to a small repeat region (~100 bp) eluding sequencing. The gap is located 2 kb in from the T7 end and has been verified by PCR using flanking primers.

Dotplot analysis of 630N19 against itself showed no extended regions of local similarity (data not shown). The overall G+C content of the complete sequence was 43.5%. Analysis of the complete sequence with Repeatmasker unveiled 2 SINEs and 69 other repeats. Repeat elements occupied 2.08% of the entire genomic sequence. Using a salmonid repeat-specific database, appr. 20% of the BAC contained repeats [29]. A total of 20 ORFs were identified within the 211 kb sequence where all but six had sequence similarity to transposable elements or other repeats.

Linkage analysis using the 3'UTR marker of classical MHC class II gene *Sasa-DAA *and a marker identified in the 630N19 BAC sequence showed that the two regions are unlinked with *Sasa-DAA *residing on linkage group 6 and the 630N19 marker on linkage group 5 (T.Moen, Akvaforsk, pers.com).

### Gene Content and Organization

**Figure 1:**
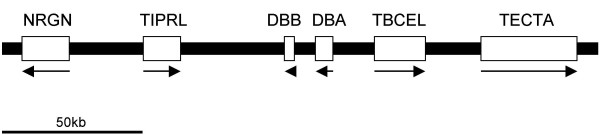
**Gene organization in the 630N19 BAC sequence**. Black line (plus boxes) shows regions of sequence information, 211190 bp in total. Locus designation is based on sequence identity to matching ESTs and human nomenclature is used. Genes are depicted by white boxes and the arrows indicate gene orientation. Sequence is drawn to scale.

The BAC encompassed six genes with homology to MHC class II alpha, MHC class II beta, NRGN (Neurogranin), TIPRL (TIP41, TOR signalling pathway regulator-like), TBCEL (Tubulin folding cofactor E-like) and TECTA (Tectorin alpha) (Fig. 1). The MHC class II alpha and beta related loci had low sequence identity to classical *Sasa-DAA *and *Sasa-DAB *sequences, and were thus designated *Sasa-DBA *and *Sasa-DBB *as suggested by R.J.M.Stet (The MHC Nomenclature Committee, pers.com). The Neurogranin domain family is the human homolog of the neuron-specific rat RC3/neurogranin gene. This gene encodes a postsynaptic protein kinase substrate that binds calmodulin in the absence of calcium [30]. The TIP41 domain family belongs to the TOR signalling pathway that activates a cell-growth program in response to nutrients in *Saccharomyces cerevisiae *[31], where TIP41 interacts with TAP42 and negatively regulates the TOR signalling pathway. TIP41 homologs are found in vertebrates, but very little information on its function is described. All the above mentioned genes have matching Atlantic salmon ESTs (Table 2).

**Table 2:** Nucleotide sequence identities between the 630N19 BAC-encoded genes and matching Atlantic salmon EST sequences.

**BAC gene**	**Abbrevation**	**EST Acc.No**.	**Identity (%)**
Neurogranin	NRGN	DY707147	100
TIP41 TOR signalling pathway regulator-like	TIPRL	DY719987	99
MHC class II beta	DBB	DY726096	100
MHC class II alpha	DBA	EG757342	100
Tubulin folding cofaktor E-like	TBCEL	CA368403	91
Tectorin alpha	TECTA	N/A	NA

**Figure 2:**
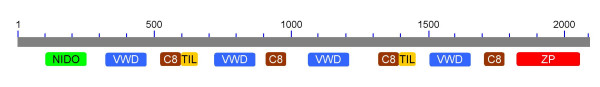
Screenshot of Blastp Domain hits for the Atlantic salmon tectorin alfa aa sequence.

Only rainbow trout EST matches were found for the TBCEL gene (Table 2). TBCEL is a novel regulator of tubulin stability with overexpression causing depolymerization of microtubules and suppression resulting in an increase in the number of stable microtubules [32]. No perfect EST match was found for TECTA, but mouse TECTA is expressed during early ontogeny and such libraries are not available for salmonids yet. The Atlantic salmon TECTA protein contained several conserved domains with identical organization to mammalian TECTA molecules (Fig. 2) [33,34]. An extracellular domain (NIDO) of unknown function is found in nidogen (entactin). The von Willebrand factor type D domains (VWD) are blood glycoprotein domains that are required for normal hemostasis. The domain of unknown function denoted as C8 contains 8 conserved cysteine residues and is found in disease-related proteins such as von Willebrand factor, Alpha tectorin, Zonadhesin and Mucin molecules. The trypsin inhibitor-like cysteine rich domains (TIL) are found in many extracellular proteins. The zona pellucida (ZP) domain is found responsible for sperm-adhesion and is present in multidomain transmembrane proteins such as glycoprotein GP2, uromodulin and TGF-beta receptor type III.

### Comparison of salmonid MHC class II sequences

**Figure 3:**
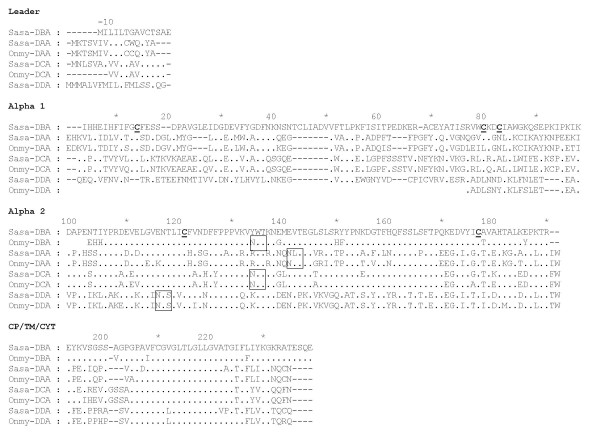
**Alignment of salmonid MHC class II alpha sequences**. Genbank accession numbers are as follows: Sasa-DAA*0101 [Genbank: L77086], Onmy-DAA*02 [Genbank: CAB96451], *Sasa-DBA *(630N19) [Genbank: EG757343], *Sasa-DCA *[Genbank: DW549478], *Onmy-DCA *[Genbank: CR376525], *Sasa-DDA *[Genbank: DW557800], *Onmy-DDA *[Genbank: BX085673]. Dots indicate identities, dashes indicate gaps or missing sequence information. Cysteine residues involved in putative Ig fold are bold and underlined, and the unique N-linked glycosylation sites found in salmonids are boxed. Individual domains and regions are defined based on mammalian class II sequences.

The *DBA *sequence showed 46% amino acid (aa) sequence identity when compared to Sasa-DAA*0101. Further UniGene and EST database searches identified one partial rainbow trout *DBA *candidate in addition to two other Atlantic salmon and rainbow trout class II alpha-like sequences which we denoted *DCA *and *DDA *(Fig. 3). Amino acid sequence identity within tentative locus ranged from 85 to 93% across species while identity between all salmonid class II alpha sequences ranged from 39 to 61% both across loci and species.

All Atlantic salmon alpha 1 domains, except *Sasa-DDA*, contained cysteines potentially involved in formation of disulphide bridges (Fig. 3). The rainbow trout *Onmy-DBA *and *Onmy-DDA *sequences are partial ESTs disrupting further comparison. A distinct feature of the *Sasa-DBA *sequence is a unique 7 aa insertion at aa position 41–47, not seen in any of the other salmonid alpha 1 domains. This insertion probably influences which cysteines are involved in formation of disulphide bridges. The salmonid alpha 2 domains contained cysteines at positions 121 and 177 potentially involved in formation of disulphide bridges. Putative N-linked glycosylation sites were found in the *DAA *(aa 141–143), *DCA *(aa 134–136) and *DDA *(aa 116–118) sequences, but not in the *DBA *sequence.

**Figure 4:**
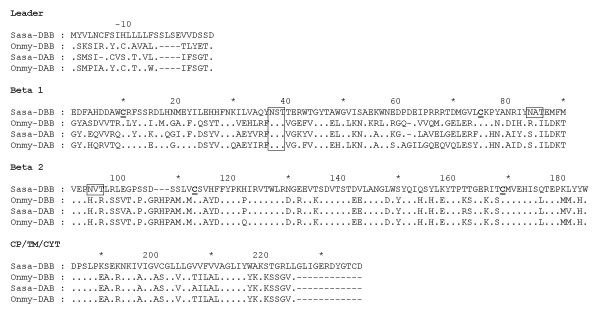
**Alignment of salmonid MHC class II beta sequences**. Sequence references are as follows: *Sasa-DBB *(630N19) [Genbank: DY726096], *Sasa-DAB *[Genbank: CAD27784], *Onmy-DBB *[Genbank: AAD53026], *Onmy-DAB *[Genbank: AAA79133]. Dots indicate identities, dashes indicate gaps or missing sequence information. Cysteine residues involved in putative Ig fold are bold and underlined, and the unique N-linked glycosylation sites found in salmonids are boxed. Individual domains and regions are defined based on mammalian class II sequences.

The *Sasa-DBB *locus showed 49% amino acid sequence identity when compared to Sasa-DAB*0201. Full-length sequencing of the *DBB*-matching EST clone [Genbank: DV106186] was performed by primer walking. Rainbow trout sequences denoted *Onmy-DBB *as locus definition name was available in Genbank, but sequence identity in the beta2 domain suggested this may be an *Onmy-DAB *allele rather than a new locus (Fig. 4). EST database searches for other salmonid *DBB*-like sequences were not successful. All salmonid beta-1 domains contained cysteines at positions 10 and 75 potentially involved in formation of a disulphide bridge (Fig. 4) and have a putative N-linked glycosylation site at aa 37–39. The *Sasa-DBB *sequence had an additionally N-linked glycosylation site at position 84–86. The salmonid beta-2 domains contained cysteines at positions 113 and 169, while the *Sasa-DBB *sequence had an additional N-linked glycosylation site at positions 94–96. Looking at the CP/CYT/TM region, the *Sasa-DBB *sequence had a 12 aa additional C-terminal sequence when compared to the other salmonid MHC class II beta sequences.

In summary, Atlantic salmon harbours at least two divergent MHC class II beta sequences (*Sasa-DAB *and *Sasa-DBB*) and four divergent MHC class II alpha sequences (*Sasa-DAA*, *Sasa-DBA*, *Sasa-DCA *and *Sasa-DDA*), which all share most of the characteristics typical for vertebrate MHC class II molecules.

### Polymorphism and expression of Atlantic salmon MHC class II molecules

To evaluate the polymorphic content of the BAC genes *Sasa-DBA *and *Sasa-DBB *we compared exon 2 sequences from 7 unrelated Atlantic salmon for both genes and to all available matching ESTs. One aa substitution was found in the *Sasa-DBB *exon 2 (G/D at pos.72), while no substitutions were found in the *Sasa-DBA *sequences. As the included sequences were derived from both Norwegian as well as Canadian waters, one may assume these genes have little or no polymorphism analogous to the nonclassical MHC class II genes identified in higher vertebrates. As for *Sasa-DCA *and *Sasa-DDA*, we have not performed any extensive polymorphic studies, but based on available EST information these loci contain little or no polymorphism

**Figure 5:**
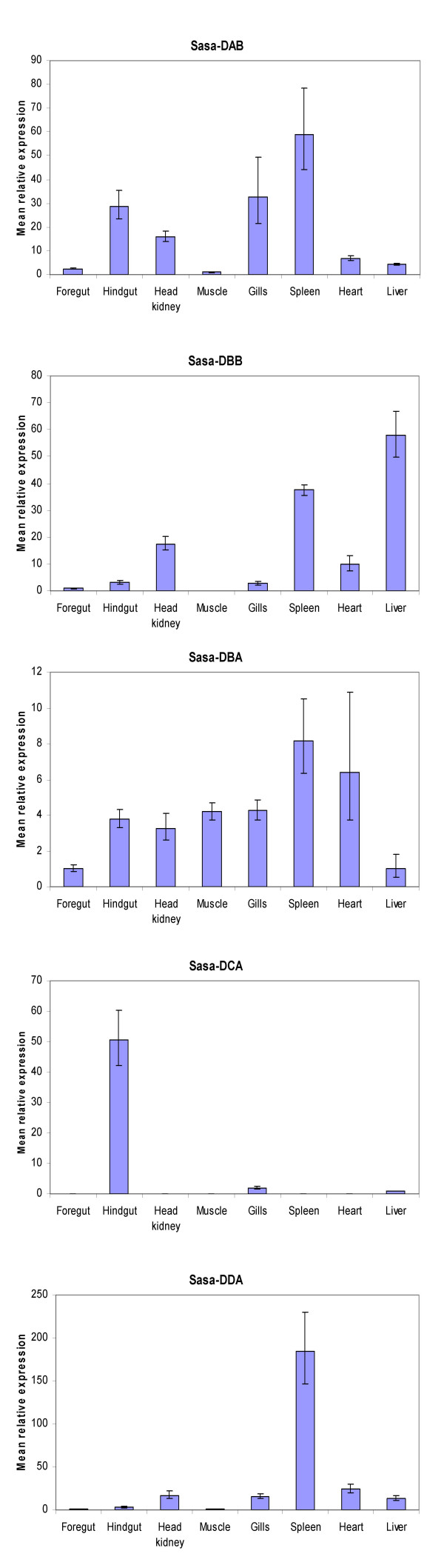
**Expression analysis of Atlantic salmon MHC class sequences**. Relative expression of *Sasa-DAB*, *Sasa-DBB*, *Sasa-DBA*, *Sasa-DCA *and *Sasa-DDA *in various tissues of Atlantic salmon using EF1A as reference gene. Data are mean ΔΔC_T _levels ± SEM values relative to the tissue with lowest expression set to one.

**Figure 6:**
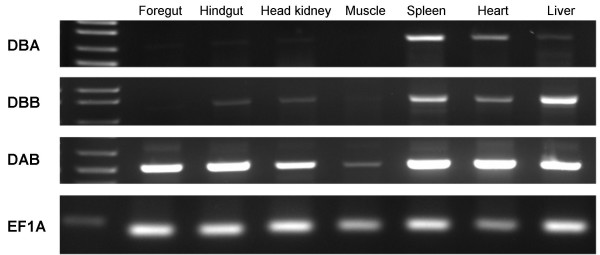
**Expression analysis of *Sasa-DBA *and *Sasa-DBB***. Semi-quantitative RT-PCR of *Sasa-DBA *(DBA19F/DBA660R) and *Sasa-DBB *(DBB98F/DBB825R) in various tissues of Atlantic salmon. Primers are located at different regions of the genes when compared to real time PCR analysis. *Sasa-DAB *was included as positive control, while EFIA was internal housekeeping control.

A common feature for all the above described salmonid MHC class II sequences is their relatively low expression, based on hits in EST databases, when compared against their classical expressed counterpart. To investigate the MHC class II expression patterns, various tissues of unstimulated Atlantic salmon were analyzed for gene expression by means of real-time PCR (Fig. 5). Foregut, hindgut, head kidney, gills, spleen, heart, liver and muscle tissues were taken from one Atlantic salmon individual, where muscle represented a non-immunologically active tissue. EF1A served as the reference gene. The highest *Sasa-DAB *expression was detected in spleen, followed by gills, hindgut and head kidney. Lower *Sasa-DAB *levels were observed in heart, liver and foregut with muscle showing the lowest expression levels. This *Sasa-DAB *expression pattern fits with the observations done by Koppang et al. [35]. *Sasa-DBB *showed highest expression in liver, followed by spleen, head kidney and heart. Our DBB-matching EST clone [Genbank: DV106186] descend from a liver cDNA library, confirming the expression of this gene in liver. Both macrophage and interhepatocytic cell populations have been found in liver of Atlantic salmon [36], suggestive of an immune function. Lower *Sasa-DBB *levels were observed in hindgut, gills and foregut with no detectable expression in muscle. *Sasa-DBA *showed highest expression levels in spleen and heart, followed by gills, muscle, hindgut, head kidney, foregut and liver. The expression patterns for the *DBA *and *DBB *genes were supported with additional semi-quantitative PCR (Fig. 6) using primers for different regions of the genes. *Sasa-DCA *showed high expression in hindgut with low expression levels in gills and liver and none in remaining tissues. Hindgut of teleosts have a well recognised capacity to take up antigen [37], an ability that has been related to the existence of a mucosal immune system in fish. *Sasa-DDA *showed a strikingly high expression in spleen. Spleen is a central organ for antigen display and response, and a major component of the fish lymphoid system. Lower *Sasa-DDA *levels were observed in heart, head kidney, gills, liver and hindgut with foregut and muscle showing the lowest expression levels. Based on raw C_T _values, *DDA *expression level in spleen was the only gene with an expression level comparable to classical DAB expression.

Based on lack of polymorphism and expression patterns deviating from their classical counterpart *Sasa-DAB*, the *Sasa-DBA*, *Sasa-DBB*, *Sasa-DCA *and *Sasa-DDA *molecules can be classified as non-classical MHC molecules similar to mammalian non-classical HLA-DM or HLA-DO molecules and can be involved in catalysis of peptide loading and stabilization of the classical *Sasa-DAA*/*DAB *molecules. However, highest expression of *Sasa-DBB *and *Sasa-DCA *in liver and hindgut respectively, and a *Sasa-DDA *expression level in spleen comparable to *Sasa-DAB*, may indicate that these molecules have a unique and yet unidentified function in these organs in salmonids.

### Phylogenetic analysis of MHC class II related molecules

**Figure 7:**
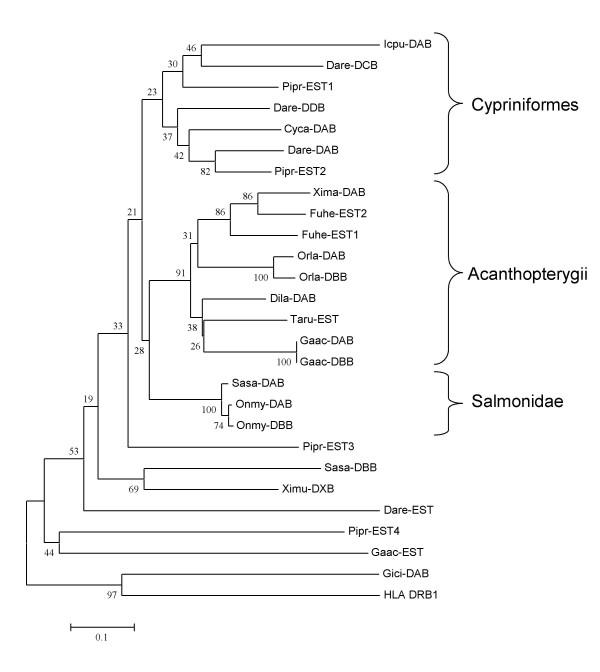
**Phylogeny of teleost MHC class II beta sequences**. Phylogenetic tree analysis by NJ method for full-length amino acid sequences. Consensus trees were based on 1000 bootstrap replications and reported with the bootstrap support values (in percent) indicated above the respective nodes. Sequence references are as follows: Sasa-DAB [GenBank: CAD27784], Sasa-DBB [GenBank: DY726096], Onmy-DAB [GenBank: AAA79133], Onmy-DBB [GenBank: AAD53026], Orla-DAB [GenBank: BAA94279], Orla-DBB [GenBank: BAA94280], Xima-DAB [GenBank: AAC05652], Gaac-DAB [GenBank: AAU01918], Gaac-DBB [GenBank: AAU01920], Gaac-EST [Genbank: DN681207], Dila-DAB [GenBank: ABH09450], Fuhe-EST1 [GenBank: CN976662], Fuhe-EST2 [GenBank: CN984097], Taru-EST [GenBank: CA846190], Icpu-DAB [GenBank: AAB67871], Cyca-DAB [GenBank: CAA64709], Dare-DAB [GenBank: NP_571551], Dare-DCB [GenBank: CAD56804], Dare-DDB [GenBank: AAA87893], Dare-EST [Genbank: CK126567], Pipr-EST1 [GenBank: DT084791], Pipr-EST2 [GenBank: DT351641], Pipr-EST3 [GenBank: DT139435], Pipr-EST4 [GenBank: DT355684], Ximu-DXB [GenBank: AAS55041], Gici-DAB [GenBank: AAF82681] and HLA_DRB1 [GenBank: AAA59781].

The phylogenetic relationship between salmon and other expressed teleosts MHC class II sequences were subjected to phylogenetic analyses. For MHC class II beta, the majority of sequences cluster according to phylogeny with zebrafish, medaka, and salmon as representatives for cypriniformes, acanthopterygii and salmonids respectively (Fig. 7). The connection between locus and expressed beta sequences are known for a few species. For medaka and stickleback, the DAB and DBB represent two very similar sequences from two different loci [12] suggestive of a recent gene duplication. The rainbow trout DBB sequence is published as *Onmy-DBB *although proof of a duplicated class II beta locus in rainbow trout is lacking. In zebrafish (Dare), authors have reported a varying number of class II loci [11,14] although the assumption has been that *Dare-DAA/-DAB *was the major expressed molecule [13]. Based on sequence divergence, the *Dare-DCB *and *-DDB *sequences most likely represent two additional expressed zebrafish class II beta loci. However, lacking knowledge on polymorphic content and expression profiles hinders definition of these genes as classical or non-classical. Fathead minnow (Pipr) contains four very divergent class II beta sequences, but their genomic organization remains to be characterized. Although difficult, some lineage-specific residues/motifs are noticeable (the sequence alignments are found in Additional File 1). The acanthopterygii class II beta sequences contain lineage specific residues such as the L215, M223, I242, L245, S248, F252, R257, R260 aa's motif in the COOH- end. Fewer distinct lineage-spesific residues exist for the cypriniformes sequences, but the S51, M53 or the I245, I247 motifs are potential candidates allowing for some exceptions. Cypriniformes as well as acanthopterygii sequences share a RILV motif at aa position 262–266.

Six teleost class II beta sequences from zebrafish, stickleback, fathead minnow, xiphophorus and Atlantic salmon (*Sasa-DBB*) identified through a thorough database search show low sequence identity to the above described sequences. No lineage specific residues are apparent between these and the above lineages although some residues shared between *Sasa-DBB *and Ximu-DXB suggest they have a common ancestor. Both these sequences share a 132–134 RLE, Y191 and T203 motif in the beta2 domain. The xiphophorus DXB sequence has been reported as a non-classical sequence [26] while none of the other EST sequences have been defined as MHC sequences in the database or in publications. Identifying expressed MHC class II molecules ease with growing number of ESTs available as exemplified by zebrafish. A total of 1.3 million zebrafish EST sequences are available in Genbank, probably explaining why we now identify four expressed MHC class II beta loci.

**Figure 8:**
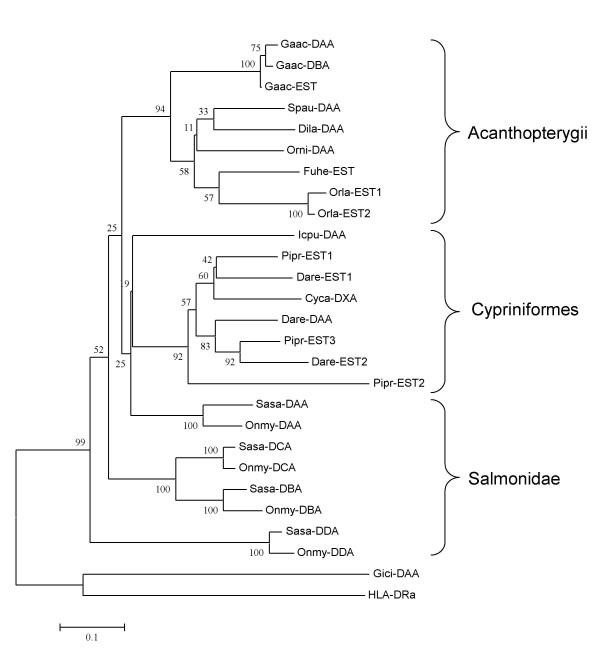
**Phylogeny of teleost MHC class II alpha sequences**. Phylogenetic tree analysis by NJ method for full-length amino acid sequences. Consensus trees were based on 1000 bootstrap replications and reported with the bootstrap support values (in percent) indicated above the respective nodes. Sequence references are as follows: Sasa-DAA [GenBank: AAL40122], Sasa-DBA [GenBank: EG757342], Sasa-DCA [GenBank: DW549478], Sasa-DDA [GenBank: DW557800], Onmy-DAA [GenBank: CAB96451], Onmy-DBA [GenBank: CX137594], Onmy-DCA [GenBank: CR376525], Onmy-DDA [GenBank: BX085673], Gaac-DAA [GenBank: AAU01917], Gaac-DBA [GenBank: AAU01919], Gaac-EST [GenBank: DN737221], Spau-DAA [GenBank: AAY42849], Orla-EST1 [GenBank: DC261023], Orla-EST2 [Genbank: BJ884671], Fuhe-EST [Genbank: CV816904], Dila-DAA [Genbank: ABH09446], Orni-DAA [Genbank: AAF66843], Cyca-DXA [GenBank: CAA64707], Pipr-EST1 [Genbank: DT253073], Pipr-EST2 [Genbank: DT092734], Pipr-EST3 [Genbank: DT311896], Dare-DAA [Genbank: NP_571565], Dare-EST1 [Genbank: CK018982], Dare-EST2 [Genbank: CO928661], Icpu-DAA [GenBank: AAD39865], Gici-DAA [GenBank: AAA49310], HLA-DRA [Genbank: NP_061984]

For MHC class II alpha, the majority of sequences also cluster according to phylogeny with medaka, zebrafish and salmon as representatives for the acanthopterygii, cypriniformes, and salmonid clusters respectively (Fig. 8). Stickleback has two reported class II alpha loci, denoted as *Gaac-DAA *and *-DAB *[12]. If the third stickleback EST sequence represents a third expressed locus or is an allelic version of the two reported remains to be established. As for the class II beta sequences, zebrafish also contains at least three different expressed class II alpha sequences most likely representing three different loci. The three different class II alpha sequences for fathead minnow is also comparable to its four different class II beta sequences. In addition to their classical DAA sequences, Atlantic salmon and rainbow trout contain three other class II alpha sequences, which are much more divergent than the different sequences identified in zebrafish or fathead minnow. The close relationship between the *Sasa-DBA *and *Sasa-DCA *sequences suggest a common ancestral gene. If *Sasa-DDA *is a more ancient duplication of the same ancestral locus, which has evolved away from the *Sasa-DBA *and *Sasa-DCA *sequences, remains to be established. The genomic location and gene surroundings of the *Sasa-DCA *and -*DDA *loci is currently unknown. As for the beta sequences, some lineage-specific residues/motifs are noticeable for the alpha sequences as well (the sequence alignments are found in Additional File 2). The acanthopterygii class II alpha sequences contain lineage specific residues such as the P82, Q107, I108, S131, V252, E269, S271 residues shared by most sequences. Lineage specific residues for the cypriniformes sequences are the D85, T154, P159, Q180, R182, V225 aa's with a few exceptions. Lacking lineage specific residues for the duplicated salmonid sequences may suggest ancient duplications or different origin.

As more ESTs are sequenced from teleosts and we move deeper into each library, more class II loci from more fish will most likely appear enabling a broader understanding of their origin and evolution.

### Genome evolution and paralagous regions

Beside the major human MHC region on chromosome 6, paralogous copies of genes from the MHC region have been found on chromosomes 1, 9 and 19 which are thought to be derived from two whole genome duplications that occurred in a common ancestor of all vertebrates [38]. Evolutionary analysis of complete genome sequences from the pufferfish species *Tetraodon *[39] and *Takifugu *[40] suggest that an additional genome duplication occurred early in the teleost lineage [350 million years ago (Mya)], close to the origin of the teleost fish themselves. All the fish of the family *Salmonidae *apparently descended from yet another unique genome duplication event that occurred in the lineage leading to salmonid fishes 25–100 Mya [41]. Evidence supporting the unique and relatively "young" salmonid genome duplication event was found by studying MHC class I regions in rainbow trout [9] and Atlantic salmon [7]. Here, class IA and class IB regions are found on separate chromosomes with a sequence identity of 85% (rainbow trout) and 82,5% (Atlantic salmon), suggestive of the duplication occurring approx. 60 Mya. Two other more distant MHC class I-like genes defined as ZE [42] and L [43] might be remnants from two of the earlier whole-genome duplication events.

The ancestral MHC must have contained both class I as well as class II. Shark and frog both contain MHC regions resembling the human MHC region containing both MHC class I as well as MHC class II molecules [1]. The MHC class II region is assumed to have been translocated to another area early in the teleost branch as teleosts are the only phylogenetic group with no linkage of class I and class II. In contrast to teleosts, shark seems to have chosen a different paralogue as its major MHC class II locus being more similar to the tetrapod MHC class II molecules than to the classical teleost class II. The salmonid DBA/DCA and DDA sequences cluster closer to the tetrapod/shark sequences and could also be a version of this paralogue. Based on the phylogenetic clustering, the salmonid DBA and DCA genes most likely originated from the whole-genome duplication event that occurred in salmonids 60 Mya, similar to the class I IA and IB region duplication. DDA and DAA might be remnants of two older whole-genome duplication events potentially analogous to the human Chromosome 1, 6, 9, 19 paralogues. The human TIPRL homolog [Genbank: NM_152902] is located on chromosome 1q23.2, a region that also encompasses MHC paralogous genes such as CD1 and COL11A1 [44] while the human homologs of NRGN, TECTA and TBCEL all reside on chromosome 11. If the salmon TIPRL and class II genes originate from the primordial human chromosome 1 remains to be established.

Other teleost genomes show some synteny to the molecules encoded on the Atlanitc salmon BAC clone. A tblastn search against Ensembl genomic databases of tetraodon, stickleback, medaka and zebrafish (Table 3) showed a tight linkage between the TBCEL and TECTA genes in all species except tetraodon. Stickleback and medaka also harbour a TIPRL homolog on the same chromosome as TBCEL and TECTA, although at a distance of approximately 12 Mb and 17 Mb away respectively. The zebrafish NRGN homolog is located on the same chromosome as TBCEL and TECTA approximately 12 Mb in distance. Homologs to the *Sasa-DBB *and *Sasa-DBA *genes were not found in any other available teleost genomes. In summary, the *Sasa-DBB *and *Sasa-DBA *genes are most likely a duplication of a primordial class II region that has been translocated into the NRGN-TIPRL-TBCEL-TECTA region. The duplication seems to be salmon specific although more teleost genome sequences may prove us wrong.

**Table 3:** Tblastn search of Atlantic salmon 630N19 BAC clone genes against available teleost genomes.

	**NRGN**	**TIPRL**	**DBB**	**DBA**	**TBCEL**	**TECTA**
**Tetraodon**	Chr.16 (0,8 Mb)	Chr.Un_random (138,0 Mb)	N/A	N/A	Chr.16 (8,0 Mb)	Chr.Un_random (82,6 Mb)
**Stickleback**	Chr.1 (8,4 Mb)	Chr.7 (12,6 Mb)	N/A	N/A	Chr.7 (24,9 Mb)	Chr.7 (24,9 Mb)
**Medaka**	Chr.13 (15,3 Mb)	Chr.14 (13,1 Mb)	N/A	N/A	Chr.14 (30,4 Mb)	Chr.14 (30,4 Mb)
**Zebrafish**	Chr.5 (69,7 Mb)	Chr.10 (24,8 Mb)	N/A	N/A	Chr.5 (57,4 Mb)	Chr.5 (55,7 Mb)

## Conclusion

In this study we describe a novel MHC class II region in Atlantic salmon consisting of two closely linked MHC class II alpha and beta loci denoted *Sasa-DBA *and *Sasa-DBB *respectively. The genes are not linked to the classical class II loci and have low sequence identity to classical sequences. The region shows some conserved synteny towards other described teleost genomic regions. Two additional divergent class II alpha sequences were also identified and represent two additional salmonid class II alpha loci. Expression patterns and lack of polymorphism make these genes non-classical class II analogues indicating that these molecules may have a unique and yet unidentified function in salmonids. In conclusion, we have identified a more complex picture for MHC class II in teleost fishes, with most species containing multiple expressed loci. These newly discovered genes may expand our understanding of MHC class II function in vertebrates.

## Methods

### BAC library screening

An Atlantic salmon (*Salmo salar*) CHORI-214 bacterial artificial chromosome (BAC) library was obtained from BACPAC Resources, Children's Hospital Oakland Research Institute [45]. The library consisted of approximately 299,000 recombinant clones, representing 18-fold genome coverage and an average insert size of 188 kb [27]. Probes specific for the *S. salar *MHC class II were PCR amplified (primers listed in Table 4) from cDNA clones (Grimholt pers com.) and purified from agarose gel slices with the GenClean III Kit (Qbiogene). Probes were radioactive labelled with α^32^P-CTP (Amersham) using Rediprime Random Labelling Kit (Amersham), including spermine precipitation of labelled DNA (Feinberg and Vogelstein, 1984). Filter hybridizations were conducted as described by CHORI. Probed BAC library filters were stored in Phospho-image cassettes for 2–24 hours and hybridizations visualized by Typhoon Phospho Image Scanner (Amersham).

**Table 4:** Primers used for probes, sequencing and gene expression.

Primer	Sequence (5'-3')	Position	Comments
oOV4F	GCGCCATACTGGACAAGACAG	Exon 2	cDNA amplification *Sasa-DAB*
oOV6R	CTGCTGCAGATTCAGCAACAT	3' UTR	cDNA amplification *Sasa-DAB*
DAB40F	ATGTCGATGTCTATCTTCTG	Exon 1	cDNA amplification *Sasa-DAB*
DAB489R	GTACCAGTCCCCGTTAGCCAG	Exon 3	cDNA amplification *Sasa-DAB*
DAA66F	TGCTGGCAGGTGTATGCAGAA	Exon 1	cDNA amplification *Sasa-DAA*
DAA660R	GAATGTTCCGGCAGCCACTCC	Exon 4	cDNA amplification *Sasa-DAA*
DBA19F	TCACTGGAGCCGTTTGCACTT	Exon 1	cDNA/gDNA amplification *Sasa-DBA*
DBA51F	CATCATGAAATTCACTTTATC	Exon 2	cDNA/gDNA amplification *Sasa-DBA*
DBA304R	CTTTGATTTTTGGTATTTTCG	Exon 2	cDNA/gDNA amplification *Sasa-DBA*
DBA660R	CCCCAGCAGCCCAAGAGTGAG	Exon 4	cDNA/gDNA amplification *Sasa-DBA*
DBB98F	CTCTGAGACTTGATTATGTAT	Exon 1	cDNA/gDNA amplification *Sasa-DBB*
DBB177F	TTGATTCCTCAGATGAAGATT	Exon 2	cDNA/gDNA amplification *Sasa-DBB*
DBB441R	CCATAAACATCTCTGTTGCGT	Exon 2	cDNA/gDNA amplification *Sasa-DBB*
DBB825R	CAGTAGATTTCGCCCAGTAGA	Exon 4	cDNA/gDNA amplification *Sasa-DBB*
DA847F	GATGGCAAAGAGGAAAGTGAG	3' UTR	FAM labeled minisatellite primer for *Sasa-DAA*
DA1054R	TTGTTATGCTCTACCTCTGAA	3' UTR	Minisatellite primer for *Sasa-DAA*
Sp6	ATTTAGGTGACACTATA	pTARBAC2.1	End sequencing BAC vector Sp6 end
T7	TAATACGACTCACTATAGGG	pTARBAC2.1	End sequencing BAC vector T7 end
M13F	GTTTTCCCAGTCACGAC	pUC19	DNA plasmid sequencing primer
M13R	CAGGAAACAGCTATGAC	pUC19	DNA plasmid sequencing primer
EF1A_F	CACCACCGGCCATCTGATCTACAA	Acc. No. AF321836	Real-time PCR Elongation factor 1-alpha
EF1A_R	TCAGCAGCCTCCTTCTCGAACTTC	Acc. No. AF321836	Real-time PCR Elongation factor 1-alpha
DAB566F	ATGGTGGAGCACATCAGCC	Exon 3	Real-time PCR *Sasa-DAB*
DAB620R	CTCAGCCTCAGGCAGGGAC	Exon 4	Real-time PCR *Sasa-DAB*
DBB418F	GCCATATGCTAATCGGATCTAC	Exon 3	Real-time PCR *Sasa-DBB*
DBB500R	ACACACAAGGCTGGAGTCACT	Exon 4	Real-time PCR *Sasa-DBB*
DBA538F	GCATCGCATGGGGCAAACAG	Exon 3	Real-time PCR *Sasa-DBA*
DBA641R	ATTCACAAAGCAGATGAGGGTG	Exon 4	Real-time PCR *Sasa-DBA*
DCA277F	GATTGTGTCGGGATGCCTTG	Exon 2	Real-time PCR *Sasa-DCA*
DCA357R	CTCCTCAGCCCTGGGGTAGAT	Exon 3	Real-time PCR *Sasa-DCA*
DDA225F	AGTTGCAGAGAGCCGAAGAGC	Exon 2	Real-time PCR *Sasa-DDA*
DDA317R	TTCACTTCATCCTTGGCATACA	Exon 3	Real-time PCR *Sasa-DDA*

### Characterization of positive BAC clones

DNA from positive BAC clones was isolated from 3 ml LB overnight cultures, supplemented with 20 μg/ml chloramphenicol, as described by CHORI. Mapping of BAC clones was performed by restriction fragment analysis with *Not*I and *Nru*I (NEB). Secondary screening of positive BACs were done by Southern blot hybridization of digested DNAs using *DAA *and *DAB *cDNA probes, in addition to end-labelled Sp6 and T7 oligos. End-labelling was performed with γ^32^P-ATP (Amersham) using T4 Polynucleotide Kinase (NEB) and hybridized overnight at 42°C with Church hybridization buffer. Filters were washed 3 × 30 min with 3 × SSC/0.1%SDS at 42 °C and visualized by Typhoon Phospho Image Scanner (Amersham).

### BAC subclone library and sequencing

The 630N19 BAC clone positive for DAA was subjected to nucleotide sequencing using a shotgun strategy. BAC DNA was isolated and purified by an alkaline lysis procedure using Nucleobond columns (BD Biosciences ClonTech). 15 microgram (or more) of isolated DNA was nebulized (Invitrogen) (20PSI/15s), trimmed and end filled with Mung Bean Nuclease (NEB), T4 DNA polymerase (NEB) and Klenow Fragment (NEB). The blunt-ended DNA was size fractioned by electrophoresis and the fragments corresponding to 1–4 kb were excised and gel purified using GeneClean III (Qbiogene). Fragments were ligated into a pUC19/*Sma*I vector (Fermentas) and transformed into XL-Gold competent cells (Stratagen) using the manufacturer's protocol. DNA plasmid from BAC subclones were isolated using standard alkaline lysis method [46]. More than 3000 BAC subclones were sequenced both using universal forward and reverse M13 primers with Big Dye Terminator Kit version 3.1 (Applied Biosystems) and analysed on ABI 3100 DNA automated sequencer. Sequences were screened for *E. coli *content using Pregap4 [47,48]. The resulting ~5500 high quality sequence reads were basecalled using PHRED [49,50], assembled using PHRAP [51] and then viewed and edited using Consed [52]. Remaining gaps or areas of uncertainty were analyzed by designing new internal sequencing primers via Autofinish [53] within Consed, followed by direct sequencing of shotgun clones containing segments of interest. Restriction digest of the isolated BAC were compared to in silico digests for assembly conformation. BAC 630N19 was deposited in Genbank under the accession number [Genbank: EU008541].

### Gene predictions and phylogenetic analysis

DIGIT [54] and GENSCAN [55] were used to predict novel genes and to identify open reading frames. Dotter [56] was used to compare the BAC sequence to itself as well as to other BACs and to identify duplicated regions. Blast searches identified possible functions of predicted genes [57,58]. EST cluster consensus files were obtained from GRASP EST database [59,60] and other EST were identified with UniGene [61,62]. Full-length sequencing of the EST cDNA clone DV106186 was performed by primer walking. Sim4 [63] and Spidey [64] were used to adjust exon and intron boundaries aligning EST/cDNA sequences to the BAC sequence. Repeatmasker [29,65] was used to identify repeats. Multiple sequence alignments of the assumed or verified expressed exons were done using ClustalX [66] followed by manual inspection.

Phylogenetic trees were created using neighbour-joining method in MEGA3 [67]. Consensus trees were based on 1000 bootstrap replications and reported with the bootstrap support values (in percent) indicated above the respective nodes. Gaps were removed and phylogenetic data reported using the Poisson correction model with uniform rates across all sites.

### Gene expression analysis

mRNA was extracted from Atlantic salmon tissues (foregut, hindgut, head kidney, muscle, gills, spleen, heart and liver) using QuickPrep *micro *mRNA Purification Kit (GE Healthcare Life Science). 1 μl of mRNA sample was used for quantification with Nanodrop spectrometer (Nanodrop Technologies, DE). All samples were DNase treated using Turbo DNA-free™ (Ambion, Austin, TX, USA). Gene specific PCR primers (Table 4) were designed manually for the amplification of approximately 100 bp fragments and synthesized by ProOligo (Paris, France). mRNA was treated with DNase and amplicons were, when possible, placed over introns and product size and specificity was confirmed by agarose gel electrophoresis (Gel logic 200 Imaging system, Kodak) and sequencing. Quantitative real-time PCR was conducted on a Chromo 4 Real-time Detection System (MJ Research, USA). Reactions were performed in 25 μl including ~10 ng of mRNA with a one-step real-time RT-PCR kit according to the manufacturer's instructions (RealQ RT-PCR Master Mix One Step, Ampliqon). PCR parameters were 55°C for 30 min, 95°C for 15 min, followed by 40 cycles consisting of 95°C for 15s, 60°C at 30s and 72°C at 30s. A melting-curve analysis was performed for each sample to check for unspecific amplification. Baseline level and cycle threshold (C_T_) were set manually and C_T _values > 37 were rejected. Relative expression of mRNA in relation to the housekeeping gene elongation factor 1α (EF1A) was calculated using the ΔΔC_T _method [68]. Data from real-time RT-PCR are presented as the mean ± SEM of at least three independent samples and the tissue with the lowest expression was used as calibrator.

Tissue-specific expression of selected genes *Sasa-DBA *and *Sasa-DBB *were determined by gene-specific amplification including MHC class II beta (*Sasa-DAB*) as a positive reference gene and EF1A as internal housekeeping control. The primer sequences are presented in Table 4. The PCR reactions (25 μl total volume) included 20 ng cDNA, 0,4 μM of each primer, 200 μM dNTPs, 1 × buffer (Herculase 10 × PCR buffer), and 0.5 units of Herculase polymerase (Stratagene). Amplifications were conducted as follows: initial denaturation at 95°C for 3 min, 30 cycles consisting of 95°C for 20 s, 54°C for 30 s, 72°C extension for 90 s; followed by a final extension of 72°C for 10 min. PCR products were separated on 2% agarose gel stained with EtBr for visualization with Gel logic 200 Imaging system (Kodak).

## Authors' contributions

HH performed library screening, sequencing and BAC restriction mapping, annotations, gene expression analysis, sequence data analysis and drafted the manuscript. MFL performed sequencing, annotations, sequence data analysis and revision of manuscript. HGB performed library screening and sequencing. UG contributed to planning, design, direction, analysis and revision of manuscript. All authors read and approved the final manuscript.

## Supplementary Material

Additional File 1 **Amino acid sequence alignment of teleost MHC class II beta sequences**. Sequence references are as follows: Sasa-DAB [GenBank: CAD27784], Sasa-DBB [GenBank: DY726096], Onmy-DAB [GenBank: AAA79133], Onmy-DBB [GenBank: AAD53026], Orla-DAB [GenBank: BAA94279], Orla-DBB [GenBank: BAA94280], Xima-DAB [GenBank: AAC05652], Gaac-DAB [GenBank: AAU01918], Gaac-DBB [GenBank: AAU01920], Gaac-EST [Genbank: DN681207], Dila-DAB [GenBank: ABH09450], Fuhe-EST1 [GenBank: CN976662], Fuhe-EST2 [GenBank: CN984097], Taru-EST [GenBank: CA846190], Icpu-DAB [GenBank: AAB67871], Cyca-DAB [GenBank: CAA64709], Dare-DAB [GenBank: NP_571551], Dare-DCB [GenBank: CAD56804], Dare-DDB [GenBank: AAA87893], Dare-EST [Genbank: CK126567], Pipr-EST1 [GenBank: DT084791], Pipr-EST2 [GenBank: DT351641], Pipr-EST3 [GenBank: DT139435], Pipr-EST4 [GenBank: DT355684], Ximu-DXB [GenBank: AAS55041], Gici-DAB [GenBank: AAF82681] and HLA_DRB1 [GenBank: AAA59781]. Dots indicate identities, dashes indicate gaps or missing sequence information and + indicate peptide binding sites based on HLA_DRB1. Individual domains and regions are defined based on mammalian class II sequences. Click here for file

Additional File 2 **Amino acid sequence alignment of teleost MHC class II alpha sequences**. Sequence references are as follows: Sasa-DAA [GenBank: AAL40122], Sasa-DBA [GenBank: EG757342], Sasa-DCA [GenBank: DW549478], Sasa-DDA [GenBank: DW557800], Onmy-DAA [GenBank: CAB96451], Onmy-DBA [GenBank: CX137594], Onmy-DCA [GenBank: CR376525], Onmy-DDA [GenBank: BX085673], Gaac-DAA [GenBank: AAU01917], Gaac-DBA [GenBank: AAU01919], Gaac-EST [GenBank: DN737221], Spau-DAA [GenBank: AAY42849], Orla-EST1 [GenBank: DC261023], Orla-EST2 [Genbank: BJ884671], Fuhe-EST [Genbank: CV816904], Dila-DAA [Genbank: ABH09446], Orni-DAA [Genbank: AAF66843], Cyca-DXA [GenBank: CAA64707], Pipr-EST1 [Genbank: DT253073], Pipr-EST2 [Genbank: DT092734], Pipr-EST3 [Genbank: DT311896], Dare-DAA [Genbank: NP_571565], Dare-EST1 [Genbank: CK018982], Dare-EST2 [Genbank: CO928661], Icpu-DAA [GenBank: AAD39865], Gici-DAA [GenBank: AAA49310], HLA-DRA [Genbank: NP_061984]. Dots indicate identities, dashes indicate gaps or missing sequence information and + indicate peptide binding sites based on HLA_DRA. Individual domains and regions are defined based on mammalian class II sequences. Click here for file
