# Supplementary material for: Multiple expressed MHC class II loci in salmonids; details of one non-classical region in Atlantic salmon (Salmo salar)
Source: BMC Genomics. 2008 Apr 28;9:193. doi: 10.1186/1471-2164-9-193 (PMC2386828; doi:10.1186/1471-2164-9-193)
Supplement: Additional File 1 — Amino acid sequence alignment of teleost MHC class II beta sequences. Sequence references are as follows: Sasa-DAB [GenBank: CAD27784], Sasa-DBB [GenBank: DY726096], Onmy-DAB [GenBank: AAA79133], Onmy-DBB [GenBank: AAD53026], Orla-DAB [GenBank: BAA94279], Orla-DBB [GenBank: BAA94280], Xima-DAB [GenBank: AAC05652], Gaac-DAB [GenBank: AAU01918], Gaac-DBB [GenBank: AAU01920], Gaac-EST [Genbank: DN681207], Dila-DAB [GenBank: ABH09450], Fuhe-EST1 [GenBank: CN976662], Fuhe-EST2 [GenBank: CN984097], Taru-EST [GenBank: CA846190], Icpu-DAB [GenBank: AAB67871], Cyca-DAB [GenBank: CAA64709], Dare-DAB [GenBank: NP_571551], Dare-DCB [GenBank: CAD56804], Dare-DDB [GenBank: AAA87893], Dare-EST [Genbank: CK126567], Pipr-EST1 [GenBank: DT084791], Pipr-EST2 [GenBank: DT351641], Pipr-EST3 [GenBank: DT139435], Pipr-EST4 [GenBank: DT355684], Ximu-DXB [GenBank: AAS55041], Gici-DAB [GenBank: AAF82681] and HLA_DRB1 [GenBank: AAA59781]. Dots indicate identities, dashes indicate gaps or missing sequence information and + indicate peptide binding sites based on HLA_DRB1. Individual domains and regions are defined based on mammalian class II sequences. [file 1471-2164-9-193-S1.pdf]

```
                    *         20         *         40         *         60         *         80         *
                                         ⇓ Beta 1 domain
Sasa-DAB     --------MSMSI-FCVSLTLVLSI---FSGTDGYFEQVVRQCRYSSKDLQGIEFIDSYVFNKAEYVRFNSTVGKYVGYTELGVKNAEAWNKG
Onmy-DAB     --------...P.A.YIC...LW..---........H.R.T...F..E..H.M.Y.......Q...I.......RF.....H.L.......SD
Sasa-DBB     -------MYVLNCFSIHL.L.FS.LSEVVDSS.ED.AHDDAW..F..R..HNM.Y.LEHH...ILVAQY...TERWT...AW..IS..K..ED
Onmy-DBB     --------..K..R.YIC.AVA..T---LYE....ASD..TR.L...I.MH.A...Q..T...V.HL.......EF.......L...KRL.R.
Orla-DAB     ----------.DSSSL--CL.F.T----LCSA.AFM.YA.NR.EFN.S..TD..Y.Y.MYY.RK..A..S.SL........F......RF..D
Orla-DBB     ---------------------------------------------------------------------------------------------
Xima-DAB     ---------------LGW.LVIIT----INAA..FR.YSADR.VFN.T..ND.Q..R..IY..K.FL..D.NL.RL........M...RL..D
Gaac-DAB     ----------.APS.ISVSL.FIG----LHAA..FMMF.TDE.VFN.TE.KD....R.SY...K.DT..S.S...F..F..Q...I.AN...D
Gaac-DBB     ----------.APS.ISVSL.FIG----LHAA..FM.FRTFR.VFN.TE.KD..Y....F...L.LT..S.S...F..F..Y..R...Y..NN
Dila-DAB     ----------.ASS.LSFSL.FIIS---LYTA..FLNYS.DR.DFN.T.PKN..Y.R..YY..L.LL..S.S..E.............RL..D
Fuhe-EST1    -----------------------------------------------------------------------------------KFGH.GF.SQ
Fuhe-EST2    ----------MASSLTW.L.FIT----VHSAGAFM.YR.VR.VFN.SE.ND..Y....CY..K..A..S.S.........F.......R..SD
Taru-EST     ----------..SSSLRVFL.FI.----LYTAG.FQYY..SR.SFN.T..KD..YSR.VYY..LM.A..S.S...FE...KY.LFQ.DY..NQ
Icpu-DAB     ----------..KLLKIL.IVLPAV---LHTAH.N.LSQPDW.IW.KE..SDM.Y.KPLII..IK.LEY......V.......I...DRF..D
Cyca-DAB     ----------LKLLIFHPILMLSAF---TGTA...Y.YTMYE.V..TS.YSDMVYLV.LS..QVVD.QC..SAV.C.....E...Y..NF..D
Dare-DAB     ---------MYLLKPFLVILMLSTF---TGTA...YDYIKQ..F..TS.YSDMVYLA..S...VVDTQ...S...F.....Q.LIF..NF..D
Dare-DCB     ---------MQFSRVACLAMILSAL---LEKVC.NYGYLQS...VL.S-TKKV.L.F.FI...I..I.Y...DQ.I.....F.E.FV.NYKNN
Dare-DDB     ---------------------------------------------------------------------------------------------
Pipr-EST1    -------MSLPKVLSFHLILMLSAF---TGAAN...YS.WSK.IW..H..SDMVY..N.I...DVFIQ.......F.....H..Y...LR.NN
Pipr-EST2    -------MSVRKFLIFHPILMLSAF---IGTA...Y.YIMSE.F..TS.YSDMVLLQ..S...VVD.QY..SL..F.....E...Y..N...D
Pipr-EST3    -------MSVPGLWSFQHILMLSVL---TGAA...YFHQWT..I...P..SDMVY.EGFY...YPF.K.......F..F..F..RI..NY.ND
Dare-EST     --------------------MLSVF---TGAA...YSSTWS..I..YP.FRDM...VG.Y...WMDIQ.......F.....Q...F..NF..D
Pipr-EST4    -MRLHWKQNTEMRT.RLT.V..GLAFCCV.SK.V.VF.NIVE.E..KV..SDMVY.IKL...QKLLCSYD.RL......D.Y.I...DHY.SQ
Ximu-DXB     ---------MAQAQG.TVFLVLFLV---..PGGAFYLS.RER.QF..S.GHDAVLL.QVY...ILE.EY......MI....KTEAL.IIL.NN
Gaac-EST     -------MTVRPESRDHVPAAPV.VKCSAADGH...MHDDFW.NMQTARP.QV.YLVDWY...EFTMQY......WT.F.AA.LVS.AVF.GN
Gici-DAB     -LGGVASRSLWIRIPFLIAV..IFNGERD..AGAHCFDQLHR.VFN.S--KDWV.LEQ..YDQELIAYYDYNQR..IAVKAWMKS.VDR..SE
HLA-DRB1     MVCLKLPGG.CMTALT.T.MVLS.RLALAGD.RPR.LWQLKFECHFFNGTERVRLLERCIY.QE.S...D.D..E.RAVE...RPD..Y..SQ
                                                 + + +                + + +    ++         +         +   ++

                100         *        120         *        140         *        160         *        180
                                           ⇓ Beta 2 domain
Sasa-DAB     P-ELAVELGELERFCKHN-AAIYYSAILDKTVEPHVRLSSVAPPSGR-HPAMLMCSAYDFYPKPIRVTWLRDGREVKSDVTSTEELANGDWYY
Onmy-DAB     AGI.GQ.QVQ..SY....-...D..................T.....-...............Q.............................
Sasa-DBB     .D.IPRRRTDMGVL..PY-.NRI.N.TEMFM...N.T.RLEG.S.DS----S.V..VHF....H........N.E..T......DV....L.S.
Onmy-DBB     Q-.VVQMR.....L..P.-.D.H.R................T.....-..............................................
Orla-DAB     TS..S.RRAQK.TY....-ID.D.QTA.S.S.Q.R..VQ.L..SG.H-.....V..V......T...S...GKE..S......A.MED.....
Orla-DBB     --------------------------------Q.S.MIE..T.SG.H-.....V..V......T...S...GKE..S......A.MED.....
Xima-DAB     QSQI.ALKAQK.TY.LN.-VGVW.QTK.T.S.A.T...F.TT..A.H-..S..V.RV......T.K.Q.....Q..T....T.D.MED.....
Gaac-DAB     ASF.SAMKAQK.VY.LNH-VPV..T.A.T.SA..Y...H.ET..G.G-PLS..V..V......K.I.R.T....PETTG....D...D.....
Gaac-DBB     .SY.SAMKAQK.VY.LNH-VPV...NA.T.SA..Y...H.ET..G.G-PLS..V..V......K.I.R.T....PETTG....D...D.....
Dila-DAB     .S...AMRAQK.TY.LTT-VN.D.Q.V...S.K.Y...R.....A.K-..S..V..V...F..H...S.....Q..T......D...DA..F.
Fuhe-EST1    TSYIEGLKAQRDTY.LN.-VG.D.QVA.T.S...Y..IH.E..SG.GGQHG..V.GV.S....T.K.......Q..T....T....PD.....
Fuhe-EST2    .SLI.GLKAQK.TY.LNH-VTA..P.A.T.S.A.T..IY.TT..A.S-.....V.RV......N.K.S.R...Q..T....T.D.MED.....
Taru-EST     SSI.EQVRDNK..Y.Q..-IKNW..N..S.S...E.IVH..V..A.G-.....V..V......RY.K.S.Q...E..SQ.....D...D...F.
Icpu-DAB     .AFMQGLKA..DSV..N.-VGN...G..S.....Q.KVKL.KKSD.T-...T......S...PA.S......N.K.I.GG......M.D.....
Cyca-DAB     .SV.QDLKTSVDTY.RS.-.QLAD.SVR..A.Q.KIT.R.ARQAG.S-R..V......E....K.K.S.....K.MT......M.M...N.F.
Dare-DAB     QAY.HQLKAQVDT..R..-.R.WD..VR..A.L.E.TIK..RQAE..-...V.L....E....K.KMS.....K..T......M.M.D.....
Dare-DCB     TFV.VLAEFGIDNCK.IAK.L.SDGMLNHV..K.E.IIR..TEAK.N-QK.F.V.........A.KL..M.NDKK.TA....I..M.D.....
Dare-DDB     --------------------------------K.T.V....TQAN..-......I....E...RH.K.S..KG.KA.T.E....M.M.D.....
Pipr-EST1    SDI.QS.RA.V.....Y.-.E.DQRT.T..S.P.Q.K....TQAG..-...V...........R.QIS.M...KV..........M.D.....
Pipr-EST2    QAS.QQLKAQVDT..RP.-.Q.FD.NVR..A.K.E.T....KQAE.N-...V......E....K.K.S.....KLIT......M.M.D.....
Pipr-EST3    .GI.QQTRAQVDV..R..-.EVTEA.VYN...Q.R.K.R..M.GDDT-...LV...V.....EQ.K.S.YK..VVMT.........P....L.
Dare-EST     QAL.QQKKS.VDSI.R..-.E..E..VF..A.K.K.N.NL.QKGDSG-..YL.K.N......QQ.KMS..KN.K-MV.EL..S.KKP..N...
Pipr-EST4    GWKMKQRKE...TL.RA.--.RL.VNSTRRK.P.V.TVRPTKKAHYG-QLST.V.H..N...QA.NI...L..S..TG..I...FMD....R.
Ximu-DXB     .EFITH.IWKTN-L..R.---TPLAQKVLTP...Y.Q.RLEKAEYSQ-.QQ..I.........Q.........K..T......D..P..N.L.
Gaac-EST     HFDVLQRKE.RRLI.VD.-VGHALN.TE.NMAA.S...AEASGSGHN---TT.V........GR..LA.....Q..T.GA.FS.VTT..N.T.
Gici-DAB     G--AEEQYQ.AKAY.E..-IPVM.ESA.ARQ...K.TIRTKESTYPG-PS.I.I.Y.VG...AK.S....KN.QK.SDADVTV.L.S....T.
HLA-DRB1     KDL.EQKR.QVDNY.R..-YGVGE.FTVQRR...K.TVYPSKTQPLQ-.HNL.V..VSG...GS.E.R.F.N.Q.E.AG.V..GLIQ....TF
             +  + ++  +   +  ++   ++ ++

                 *        200         *        220         *        240         *        260         *
                                             ⇓ CP/ TM/ Cyt
Sasa-DAB     QIHSHLEYTPRSGEKISCMVEHISLTEPMVYHWDPSLPEAERN---KIAIGASGLVLGAILALAGLIYYKKKSSGVL--------------
Onmy-DAB     ..........K..................M.............------..........T..................--------------
Sasa-DBB     ..Q.Y.K...TT..R.T.......Q...KL.Y......KS.K.----..V..VC..L..VVFVV.....WA.STGRL.GLIGERDYGTCD--
Onmy-DBB     ..........K..................M.............------..........T..................--------------
Orla-DAB     ..................K...A..KD.L.TD....M..S...---.V.......I..LV.S...F....R.AR.RILVPSS---------
Orla-DBB     ..................K...A..KD.L.TE....M..S...---.V.......I..LV.S...F....R.AR-----------------
Xima-DAB     .V..Q.........R...R...V..K..LITD....M..S...---.L.......I..L..S...F....R.AR.RILVPTS---------
Gaac-DAB     .T................V......SK.L.TD.N..M..S...---.V.......I..LT.S...F....R.AR.RILVPSH---------
Gaac-DBB     .T................V......SK.L.TD.N..M..S...---.V.......I..LT.S...F....R.AR.RILVPSH---------
Dila-DAB     ..................V...A..R..L.TD....M..S...------.......I..L..S...F....R.AR.RILVPSN---------
Fuhe-EST1    .V..T.................A..K..LITD.N..MS.......---.L.......I..LV.S...F....R.AR.RILVPTN---------
Fuhe-EST2    .V..N.................A..K..LIT..N..MS...-------------------------------------------------
Taru-EST     .L................V...A..KT.L.KD....M..S....---Q........I..L..S...F..F.R..R.RILVPNN---------
Icpu-DAB     .V......M.E...E...V.Q.A.F.K..N.K..S.M..PDKS---.............IV.SA..F..........RILVPT----------
Cyca-DAB     ....E.....K.............A.FSK..ITD.......G...---...........I.I.A..........A.RILVPN----------
Dare-DAB     ..........K.....Q.L...A...Q.LTKD.N.HIS.SD..---.F...........I.I.I..........T.RILVPN----------
Dare-DCB     ........F.QP......V.D.A.FHK..I.Y.......T..S---..IL..V..LM.IFT.A..V....R.QT.----------------
Dare-DDB     ....E......P...........A.SS...I.D.......S...---.SLL....FGD.IHNSAV.IL..R...T.LRRLD-----------
Pipr-EST1    ..........K.......A.D.AG..K.IIVD....M..P....------........I.I.A.........IT.RILVPS----------
Pipr-EST2    ....E.....K.......V...A.SPQ.I.KD.N..M.DI...------......S......I.I.A..........RILVPN----------
Pipr-EST3    ....E......A.QQ...V...A.FSK.........PV..F.L.VELLL.VETA..M..ITT.A..F....IRTGL----------------
Dare-EST     .N..E.VFN.K...T.......S.S.RTI.IELK.PVH.V.WEFQLLS.ARD.SA...I.I.A..YFPTD---------------------
Pipr-EST4    .M....DLVLHR.VSV..R...SG.EK.L.VQ..STSLDTRIA---.L.V.CFSFL..L.V.FSAAYI.Y.RXH-----------------
Ximu-DXB     ...TY..F..KP....T..L..A..KK.NL.D.E.-E.DSKWS---..VV.SA..L..LVFSI..F....TT.N.RVVVPTTEDVCPEETL
Gaac-EST     AV..S.SF..GGRDRV..K...AG.QK.ALRT.AAHGARDWETG--FLVG.VCA.L...ACLSS...VHRR.Y.NIS-------------
Gici-DAB     .VRQY.Q.E.VY.D.YT.H...S...S..SVD.EVESTSKSEK--T..IV.VL.FGF.L.IL...V.MRL.NAKAI.DSNHGPRLMGPAVS
HLA-DRB1     .TLVM..IV.....VYT.Q...P.V.S.LTVE.RARSES.QSK----MLS.VG.F...LLFLG...FI.FRNQK.HSGLQPTGFLS-----
```
