# Supplementary material for: Multiple expressed MHC class II loci in salmonids; details of one non-classical region in Atlantic salmon (Salmo salar)
Source: BMC Genomics. 2008 Apr 28;9:193. doi: 10.1186/1471-2164-9-193 (PMC2386828; doi:10.1186/1471-2164-9-193)
Supplement: Additional File 2 — Amino acid sequence alignment of teleost MHC class II alpha sequences. Sequence references are as follows: Sasa-DAA [GenBank: AAL40122], Sasa-DBA [GenBank: EG757342], Sasa-DCA [GenBank: DW549478], Sasa-DDA [GenBank: DW557800], Onmy-DAA [GenBank: CAB96451], Onmy-DBA [GenBank: CX137594], Onmy-DCA [GenBank: CR376525], Onmy-DDA [GenBank: BX085673], Gaac-DAA [GenBank: AAU01917], Gaac-DBA [GenBank: AAU01919], Gaac-EST [GenBank: DN737221], Spau-DAA [GenBank: AAY42849], Orla-EST1 [GenBank: DC261023], Orla-EST2 [Genbank: BJ884671], Fuhe-EST [Genbank: CV816904], Dila-DAA [Genbank: ABH09446], Orni-DAA [Genbank: AAF66843], Cyca-DXA [GenBank: CAA64707], Pipr-EST1 [Genbank: DT253073], Pipr-EST2 [Genbank: DT092734], Pipr-EST3 [Genbank: DT311896], Dare-DAA [Genbank: NP_571565], Dare-EST1 [Genbank: CK018982], Dare-EST2 [Genbank: CO928661], Icpu-DAA [GenBank: AAD39865], Gici-DAA [GenBank: AAA49310], HLA-DRA [Genbank: NP_061984]. Dots indicate identities, dashes indicate gaps or missing sequence information and + indicate peptide binding sites based on HLA_DRA. Individual domains and regions are defined based on mammalian class II sequences. [file 1471-2164-9-193-S2.pdf]

```
                                           ⇓ alpha 1 domain starts
                 *         20         *         40         *         60         *         80         *
Sasa-DAA   -------------MKTSVIVLILCWQVYAEHKVLHIDLVITGC---SDSDGLDMYGLDGEEMWYADFNKQEG-------VVALPPFADP--FTFP-
Onmy-DAA   -------------....M......C.....D....T.IY.S..-------....V.........L.......K..-------........Q--IS..-
Sasa-DBA   ------------------MI...TGA.CTSAEIH.EIHF.F..FES.--.PAVGLEI..D.VF.G....NSNTCLIADV.FT..K.ISITPEDKER
Onmy-DBA   -------------------------------------------------------------------------------------------------
Sasa-DCA   -------------.NL..AIVV.TAV.CTSAEIP.ETVYVL..LEKTKVKAEAELQ.....VV....QSGQE-------.WT..E.LG.FSSSTVR
Onmy-DCA   --------------------------------------------DTKVEAEAELQV....VV....QSGQE-------.WT..E.LG.FPSSTVR
Sasa-DDA   -------------.MMALVFM..LFMLSSTQGQEQHVFNVINTR-SETEEFNMTIVV.DN.YLHVYL.NK..--------..T..EWGNY-------
Onmy-DDA   -------------------------------------------------------------------------------------------------
Gaac-DAA   --------MKTKT.MKMMV..V.SGVFCVSADG--E.IA......------....E.........L.....KHGK.--------.KPQ.S.V..--IE.QE
Gaac-DBA   --------MKTKT.MKMMV..V.SGVFCVSADG--E.FA......------....EF........I.....KHGK.--------.KPQ.S.V..--IDYQE
Gaac-EST   MVLVHRELMKTKT.MKMMV..V.SGVFCVSADGP.E.IA.D..------....EM........V.....KHGK.--------.RP..S.V..--ID-NP
Spau-DAA   -------MMKMMK.MKMMV..V.SCVLCVSAE...Q.IAVV..----.A...EMV......V......KNKK.--------.YPV.D.VG---IRYQE
Orla-EST1  ---------------------------------------------------..A.....VY......KTI-------.EPQ...V.R--TSLAE
Orla-EST2  --------------MKMKLL.FV.GILSGTAA.F.E..A........------....E...A.....VV.....K..T.-------.EPQ...V.H--VSY.-
Fuhe-EST   ----------------MRLL.F..GVLRVSAD...E..N.V.--------....EF...A.......F....KQEK.-------.EPQ...I.H--ISYRE
Dila-DAA   -------------..MMMMMVLVL.FSCVSAD...E.IS.N.--------....EP........I.....INKK.--------.EPQ.N.I.H--MSLQE
Orni-DAA   ---------------MKELL.F.SCVLCVSADGQ.T.IN.V..----..F..EV.........K.......HI.--------.YPQ...VVNP-.HYQE
Cyca-DXA   ---------------MYGVL.M.ALI.ST.TQ.VNR.VQFV..----..TEREFLI.F....L.H...IRK..--------..TV.D.....--IG..-
Pipr-EST1  -------------.EMYGVL.M.AVI.RT.TQ.V.R.VGFV..----..TEKEYLM.F....IFH...IRKD.--------.MT..D.....--ISN.-
Dare-EST1  -------------.DLFGFL.TFTVILSNVAQAE.R.VDFF..----..TEKEYLQ.F....LYHS..IRKV.--------..TA.D.....--MSY.-
Pipr-EST2  ---------------MELAL.T.TVLLSTDAQFI.NYFY......----.GTEEES.L.S......H...IRKM.--------.NT..D.....--MS..-
Dare-EST2  ----------MRK.ELY.LISMFTVL.SSDN..V.E.FYLL..----.NTEREY...M....LF......GVA-------.D...D.....--I.K.-
Pipr-EST3  -------------.EL..FMFM.VAL.SS.N..V.E.F.LK..----.NT.KEF...V....RI....V.GVA-------.DS..D.VEH--PSV.-
Dare-DAA   -----MFFLALRK.EVY.FI.T.SVF.SS.VN.V.E.I.MD..----..TEKEYISV......YHT..SGKR.-------EMT..D.......--.Y.-
Icpu-DAA   -------------.RLFLLCFT.VCVKDT.AQIK.H.IKVIA.----..T.KEYVL.I.K..VY....V..LI-------.K.......T--LNP.E
Gici-DAA   -----------MEARNYFS..V.VLIQGGWAGKYLY.FTQVYFVQQRSPEKHFDVME..D.IF.M...LKKE-------.ARI.E..HL----YMQ
HLA-DRA    ------MAISGVPVLGFF.IAV.MSAQESWAIKEEHVIIQAEFYLNP.QS.EF.FDF..D.IFHV.MA.K.T-------.WR.EE.GRF----ASF
                                                 + + +          + + +    ++                 +        +     ++

                                          ⇓alpha 2 domain starts
              100         *        120         *        140         *        160         *        180         *
Sasa-DAA   GFYEQAVGNQGVCKGNLAKCIKAYKNPEEKIDPPHS-SIYPRDDVDLGVENTLICHVSGFFPAPVRVRWTRNNQNLTEGVRLSTPYPNADFTLNQF
Onmy-DAA   ........DLEIL..............P.T......-........E.E.K............P...........V...G.I......T.V.F...
Sasa-DBA   -AC.Y.TISRVW..DCI.WGKQSEPKIPKIK.A.EN-T......E.E..........F.ND...P..K.Y..K.EMEV...LS..RY...K.G.FH..
Onmy-DBA   ------------------------------------E-HH....E.E..........F.ND...P..K.N..K.GMEV...LS..HF...K.G.FH..
Sasa-DCA   N..KN..KGRRL.RDA..LW.FEE.S.P.VK.A.E.-T....AEEE.........FANH.Y.P..K.N..K.GLEV...TS..RY...E.G.FH..
Onmy-DCA   N..KN..KGRRL.QDA..LW.LEE.C.P.VK.A.E.-T....AEEV.........FANH.Y.P..K.N..K.GLEV...AS..RY...K.G.FH..
Sasa-DDA   VDCPIC.RVAESRRAD.NND..LFNLETPEAKV.PEIKL.AK.E.K..IN.S.V.F.NN...P..Q.K..K.DE.VPK..KVGQYAT.S.Y.FYR.
Onmy-DDA   -------------RAD.SNY..LFSLETPEAKV.PEIKL.AKEE.K..IN.S.V.FINN...P..Q.K..K.DE.VPK..KVGQYAT.S.Y.FYR.
Gaac-DAA   .T..L.....QI.RI..KNRL.GL.DVPLEK...S.HM...K.G.E..EK.S......T..Y....TFS..K.QE.VN..SSRNV.F..N.G.F...
Gaac-DBA   .A..S..A..QI.RN..KTHV.VF.DVPLEK...S.HM...K.G.E..EK.S......T..Y....TFS..K.QE.V...SSRNV..L.N.G.F...
Gaac-EST   .G..S..A..QI.RN..KNAV.GL.DVPLEK...S.HM...K.G.E..EK.S......T..Y....TFS..K.QE.V...SSRNV....N.G.F...
Spau-DAA   .A.Q..EA.LQI..T..DIVRQ...DVPPER.A.S.PV.....E.E.E.K.I......T..Y....KFS..K.GK.V...TSVNV..M.K.G.Y...
Orla-EST1  .G..N..A..QI.RQ..KVTRE.T.GLPL.R...SNVVV.N.NK.E..EQ..........Y....N.S..K.GERVSG--SINI.F.SS.G.FT.I
Orla-EST2  .G.....A..QI.RS..KISRI.M.DLPLER...SNVVV.S..E.E..EQ..........Y....N.S..K.GERVSG--SINI.F.SS.G.FT.I
Fuhe-EST   .T..G..A.LQI.R...NV.RQTM.GIP.QK.G.SGLV..T..E.E..EK.I.V......Y....N.S..K.G.KV...TSINV.F.SK.S.FT.I
Dila-DAA   .A..G..A..QI.RQ..EIRR.SI.D-PL.F...S.PV..T..N.E..EK.......T..Y....K.Y..K.GK.V...TSINV....K.GSFT.T
Orni-DAA   .T..G..T.VQ..QNA.KIVRE.M.DFPP.HIA.SAVM..T..E.EF..Q.I......T..Y....N.S..K.G.KV...SSINV..I.K.GSFK.T
Cyca-DXA   ....TG.ALME...Q...LN..V..PTD.QLA..DA-.V.SEG..V...Q.......T.L..P..N.S..K...IV..D.S..QYRRKN.G.F.I.
Pipr-EST1  .G..NG.A.IE...Q..DVD..V..PSV.QL.I.DT-....KAI.VQD...K......T....P..N.S..K...IV...MS..QYRRKD.G.F.I.
Dare-EST1  ....NS.AQME...QD..TD....NS...QL...VT-....SE.E.V.DET.......T....P..N.S..K..DIV..EISF.QYRR.S.G.F.M.
Pipr-EST2  ....AS.AQ.E...Q..DV.....N..P.EL...ET-....QN..QP....A......T....P..N.S..K..VMV....S..QSR.RT.GLFHV.
Dare-EST2  .M.D.GL.DVQ...S..DVA.....S.Q.....K.QT-..FAENE.Q.ND..I......T....P.....S..M..KA.P.-HS..QYR..D.G.Y.I.
Pipr-EST3  ...DS.SA.IQI.LA..DVA........Q..M.K.QT-...TKN..Q.N...V......T....P.....S..K..EVFK.-DS..QYR..D.G.Y.I.
Dare-DAA   .T...SLADYET..H..DVAA....S.L..L...QT-...S....QPDI..K......T....P.....S..K..EIV...MSV.QYR..N.G.Y.I.
Icpu-DAA   TA.QS.GAEIDI..T..GVYRTEF.DTPTPQ.V.QN-......V..N..SK......STR...PTI.IT..K.GVDV.DESS..QY....E.N.Y...
Gici-DAA   .GEAAISA.IAIV.N..KVDMNLSAGTP.PKVR.EV-.V.SE.L.EW.QL.....FAD..Y.PHITMK.R....EPM.D.DNITEF.IKE....FRR.
HLA-DRA    EAQGAL-A.IA.D.A..EIMT.RSNYTPITNV..EV-TVLTNSP.E.REP.V...FIDK.T.PV.N.T.L..GKPV.T..SETVFL.RE.HLFRK.
           +  +    +  +  ++  +   +

                                                ⇓ CP/ TM/ CYT
                200         *        220         *        240         *        260         *        280
Sasa-DAA   SSLPFTPEEGDIYGCTVEHKGLAE-PLTRIW-----EPEVI---QPSVGPDVFCGVGLTLGLLGVAAGTFFLIKGNQCN-----------------
Onmy-DAA   ...S..................T.-......-----....S---....A.A..............T............-----------------
Sasa-DBA   ...S...QKE.V.I.A.A.TA.K.-.K..--------.YK.SGSS-AGP..A..............T.I.LIY..KRATESQE-------------
Onmy-DBA   ...S...QKE.V.I...A.TA.KY-.K..--------.YK.SGSS-VGP..AI.............T.I..IY..KRATESQE-------------
Sasa-DCA   ...S...Q...V.....K.TA.ED-.K..F.-------Y..REVSGS.A..A...............T...LYV..Q.F.-----------------
Onmy-DCA   ...S...Q...V.A.....TA.ED-.K..F.-----YKIHEVSGS.A..A...............T...LYV..Q.F.-----------------
Sasa-DDA   .T.T.E.Q.....T.I.D.TA.D.-.....T.------.F..PP--RA....A....L.........VP.....V..T..Q-----------------
Onmy-DDA   .T.T.E.Q.....T.I.D.MA.D.-.....T.------.F..PP--H.....A....L..........T.I...V..T.RQ-----------------
Gaac-DAA   .T.E...KL....S.M....LA.DH-..VS-------------------.....A........V...............E.S-----------------
Gaac-DBA   .T.E...KL....S.M....LA.DH-..VSFLSP---DVQ.S---.......A........V...............E.S-----------------
Gaac-EST   .T.E...KL....S.M....LA.DH-..VKFY-----DVQ.S---.......A........V...............E.S-----------------
Spau-DAA   .K.D...QQ..F.S.S.S.PA.KD-.........-----DV.KT---L.G...A....L...V...............E.R-----------------
Orla-EST1  .R.Q.V.QL....S.S...PA.T.-VQ.K..------DV.KT----..G...A........AV...............E.S-----------------
Orla-EST2  .R...V.QL....S.S...PA.T.-VQ.K..------DV.KT----..G...A........AV...............E.S-----------------
Fuhe-EST   .R.D.I.QL....S.S.D.PA.QK-..........-----DV.LDS-P......A....L...I.......V......Q..E.S-----------------
Dila-DAA   .R.E.I.QL..M.S.S.K.LS.KD-.....F.------DV.KP---E..I..A....L...V...............E.S-----------------
Orni-DAA   .R.D...QL..M.S.....VS.T.-...K.Y-----DVDSSGQSD.....AA.......V...............E.S-----------------
Cyca-DXA   ...K...A.....S...Y..A.ESRFI.KT.EV---DVA.P-----G...A.......S...............L.N..-----------------
Pipr-EST1  .T.K...K.....S...N..SIPGQ.I.KT.DV---DVALP-----..............................N..-----------------
Dare-EST1  .A.K...A.....S...N.RSIQGP.N.KT.EV---DV.LP--------...A.......V.........HSPHKETLH------------------
Pipr-EST2  ...KII..HR...S...N.RA.QG-.Q.K..GVGKASAVAP------.A..VL...L.M..A...M.T.IC.C..ATATDHSDLAKKEKKAMQWIYQ
Dare-EST2  ...K...Q.....S.S.N.TS.EQ-LQ.KT.EV---.AAMP-------....A.......A.........--------------------------
Pipr-EST3  ...K...V.....S...N.TS.E.-.K.KT.DV---.VA.P--------...A.......S...............N..-----------------
Dare-DAA   .T.R...V.....S.S.N..T.EQ-.Q.KA.EV---.VAMP-------....A.......F...............N..-----------------
Icpu-DAA   .H.....K...V.T...Q.EA.QT-.D..T.EV---DVDLP-------....A.......AV......T.....V......-----------------
Gici-DAA   .Y.SIV.SP..M.S.H...SS.QD-.V.VF.-----DQG.P-EEK-.GHGTII.AL.....IISAVV.IIL...-ERQRLQAQQHGI---------
HLA-DRA    HY...L.STE.V.D.R...W..D.-..LKH.------.FDAP-SPL.ETTEN.V.AL...V..V.III..I.I...LRKSNAAERRGPL--------
```
